# Opportunistic Detection of Lumbar Scoliosis on DXA Images in Postmenopausal Women

**DOI:** 10.3390/diagnostics16121878

**Published:** 2026-06-17

**Authors:** Kasidech Suwanpidok, Chanika Sritara, Wichana Chamroonrat, Sasivimol Promma, Arpakorn Kositwattanarerk, Chaninart Sakulpisuti, Kanungnij Thamnirat

**Affiliations:** 1 Division of Nuclear Medicine, Department of Diagnostic and Therapeutic Radiology, Faculty of Medicine Ramathibodi Hospital, Mahidol University, Bangkok 10400, Thailand; kasidech.tez@gmail.com (K.S.); chanika.sri@mahidol.ac.th (C.S.); wichana.cha@mahidol.ac.th (W.C.); sasipromma@gmail.com (S.P.); arpakorn.kos@mahidol.ac.th (A.K.); chaninart.sak@mahidol.ac.th (C.S.); 2 Division of Nuclear Medicine, Department of Radiology, Chonburi Cancer Hospital, Chonburi 20000, Thailand

**Keywords:** lumbar scoliosis, postmenopausal women, dual-energy X-ray absorptiometry, Cobb angle, bone mineral density

## Abstract

**Background**: This study aimed to determine the prevalence of DXA-detected lumbar scoliosis in postmenopausal women based on dual-energy X-ray absorptiometry (DXA) scans and identify associated risk factors. **Methods**: A total of 261 postmenopausal women aged ≥50 years who underwent lumbar spine DXA before June 2021 were included. Lumbar scoliosis was defined as a Cobb angle ≥ 10° measured from DXA images. Logistic regression analysis was performed to evaluate associated risk factors. Diagnostic performance of DXA-based Cobb angle measurements was assessed in the radiographic validation subgroup using radiography as the reference standard. **Results**: The prevalence of DXA-detected lumbar scoliosis was 14.9% (39/261; 95% CI, 10.8–19.9%). Increasing age was significantly associated with scoliosis, while body mass index, bone mineral density, and T-scores at the lumbar spine, hip, and femoral neck were not. DXA and radiographic Cobb angle measurements demonstrated strong agreement (ICC = 0.91, 95% CI 0.73–0.96), with a mean difference of −2.63°. Diagnostic accuracy was 82.1%, with sensitivity 62.1%, specificity 97.4%, PPV 94.7%, and NPV 77.0%. ROC analysis demonstrated good discriminative performance (AUC = 0.88, 95% CI, 0.79–0.98); an exploratory cutoff of 6.5° yielded the highest Youden index. **Conclusions**: DXA-detected lumbar scoliosis was identified in 14.9% of postmenopausal women undergoing DXA. DXA-based Cobb angle measurements demonstrated strong agreement with radiographic assessment and may facilitate opportunistic case detection of likely lumbar scoliosis during routine BMD assessment.

## 1. Introduction

Adult scoliosis is a spinal deformity occurring after skeletal maturity and is defined by a Cobb angle of at least 10 degrees [1]. It primarily encompasses two forms: idiopathic scoliosis, which originates during adolescence, and degenerative scoliosis, which typically develops after the age of 50 due to progressive spinal degeneration [2,3]. The condition poses increasing public health concerns, as its prevalence rises substantially with age and is consistently reported to be more common in females [4,5,6]. Although early stages may be asymptomatic, progression can lead to chronic back pain, postural imbalance, and visible deformity, ultimately impairing quality of life and increasing healthcare utilization [7,8,9,10,11,12,13,14,15].

Dual-energy X-ray absorptiometry (DXA) is typically used for osteoporosis assessment, yet lumbar spine DXA images can also be utilized to measure Cobb angles and evaluate spinal alignment [9,15,16]. This dual function has attracted growing research interest, particularly because DXA offers low radiation exposure, widespread availability, and routine use in older adults. However, despite these advantages, the potential role of DXA images for opportunistic identification of scoliosis in adults is not yet well established, and further evidence is needed to clarify its diagnostic value in this population.

Recent research on adolescent idiopathic scoliosis has shown that the severity of spinal deformity may be associated with decreased bone mineral density (BMD) [17,18,19,20] and lower body mass index (BMI) [21]. However, whether these associations are also present in adult or degenerative scoliosis remains uncertain, and studies examining prevalence and associated factors in older adults remain limited.

Several epidemiological studies have explored the prevalence and associated factors of scoliosis in adults across various populations. Schwab et al. [8], in a prospective study of adults over 60 years in the United States, reported a scoliosis prevalence of 68% based on whole-spine radiographs, with no significant sex differences and no association between age and curve magnitude. In a large Korean cohort, Hong et al. [4] found a prevalence of 35.5% among individuals over 60 years, with higher rates in females and increasing prevalence and Cobb angle severity with advancing age. Studies using lumbar spine DXA have demonstrated somewhat lower prevalence rates. Urrutia et al. [9], in Chilean postmenopausal women aged ≥ 50 years, reported a prevalence of 12.9% and found a positive association between increasing age and lumbar scoliosis, whereas bone mineral density was not associated with scoliosis. Similarly, Xu et al. [10] studied Chinese adults over 40 years and found a prevalence of 13.3%, with rates highest in the 81–90-year age group and more common in females. More recently, Rubin et al. [7] reported a prevalence of 25.2% in U.S. postmenopausal women aged 64–99 years using lumbar spine DXA, noting that both osteoporosis and Cobb angle severity increased with age, although T-scores at the spine, femoral neck, and total hip were not associated with Cobb angle magnitude.

Although these studies provide valuable insight, evidence remains limited regarding the prevalence of lumbar scoliosis among postmenopausal women and the factors associated with its development when using DXA imaging. Moreover, the diagnostic validity of Cobb angle measurements derived from DXA compared with standard spine radiographs requires further clarification.

Given these gaps, the present study aims to evaluate lumbar scoliosis on lumbar spine DXA images in postmenopausal women. The primary objective is to determine the prevalence of DXA-detected lumbar scoliosis and identify factors associated with lumbar scoliosis in this population. In addition, this study evaluates the validity of DXA-based scoliosis assessment by comparing Cobb angle measurements from lumbar DXA images with those obtained from standard spine radiographs.

## 2. Materials and Methods

### 2.1. Study Populations

This study utilized a cross-sectional descriptive design. Eligible participants were consecutive postmenopausal women aged 50 years and older who underwent BMD assessments using DXA (Discovery A with Apex Software version 13.4.2 and Horizon A with Apex Software version 13.6.0.7; Hologic Inc., Bedford, MA, USA) before 1 June 2021, at a university hospital in Bangkok, Thailand. Participants were retrospectively identified from the institutional DXA database until the predetermined sample size was achieved. Postmenopausal status was determined from the medical records at the time of DXA examination. For participants with multiple eligible DXA examinations, only the first examination that met the study eligibility criteria was included in the analysis. The exclusion criterion was individuals with a history of spinal surgery involving instrumentation, as determined through questionnaires or radiological evidence. Data were collected on demographic factors, including age, weight, height, and BMI, along with bone density values at the lumbar spine, hip, and femoral neck.

**Figure 1:**
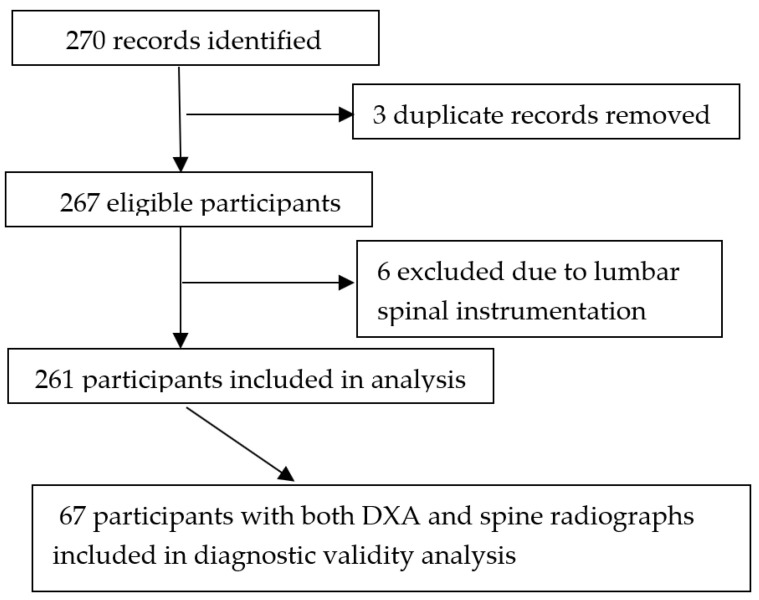
Flow diagram of participant selection and subgroup inclusion for diagnostic validity analysis.

The required sample size for estimating the prevalence of lumbar scoliosis was calculated using an expected prevalence of 12.9% based on Urrutia et al. [9], a margin of error of 0.04, and α = 0.05, yielding an estimated sample size of 270 participants. Three duplicate participant records were identified and removed during data verification. A total of 267 eligible participants were identified, and after exclusion of 6 participants with lumbar spinal instrumentation, 261 participants were included in the final analysis (Figure 1).

### 2.2. Assessment of Scoliosis

To assess scoliosis, the Cobb angle was measured by drawing lines along the upper and lower endplates of the vertebrae that demonstrate the most tilt. The angle formed by these lines indicates the degree of spinal curvature, with angles of 10 degrees or more classified as scoliosis [1,22,23,24].

**Figure 2:**
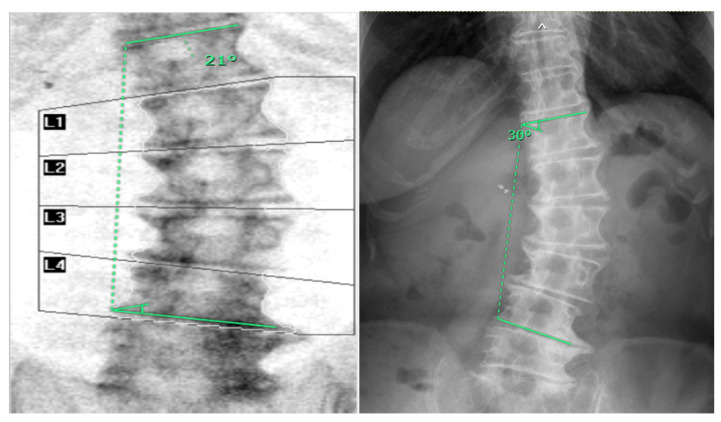
Measurement of the Cobb angle from lumbar DXA images and spine radiographs.

This study evaluated the lumbar spine DXA scans from all participants, collecting radiological parameters including the Cobb angle, curve apex, curve direction, and vertebral rotation, as illustrated in Figure 2. The curve apex refers to the vertebra or intervertebral disc with the greatest rotation or the furthest distance from the center of the spine. Curve direction is assessed based on the convex side of the spinal curvature. Vertebral rotation is evaluated using the Nash–Moe method, where the convex side of the curvature is divided into three segments, and the position of the pedicle is assessed across five levels. To assess measurement reliability, 30 lumbar DXA images were selected using a systematic sampling approach for repeated Cobb angle measurements. Intraobserver reliability was assessed by repeated measurements performed by the principal investigator at least 1 month apart without access to previous results. Interobserver reliability was independently assessed by the principal investigator and supervising physician. To assess the reliability of radiographic Cobb angle measurements, a subset of 30 radiographs from the validation cohort was independently measured by a second reader blinded to the original measurements, clinical information, and DXA scoliosis classification. Interobserver reliability was evaluated using the intraclass correlation coefficient (ICC).

### 2.3. Validity Study of Scoliosis Assessment

Among the 261 participants in the main cohort, 67 had both lumbar DXA images and corresponding spine radiographs available and were included in the diagnostic validity analysis. The validity study involved analyzing Cobb angle measurements obtained from both lumbar DXA images and spine radiographs, which served as the reference standard for scoliosis assessment. The time interval between the two assessments was no more than 12 months, regardless of the sequence of scans. Among the 67 participants included in the diagnostic validity analysis, the mean interval between DXA and radiographic examinations was 85.9 ± 87.2 days (median 49 days, interquartile range 16–134 days; range 0–343 days). Although some degree of progression may occur over time, degenerative lumbar scoliosis generally progresses slowly, and measurable progression often requires several years of follow-up [3,14,25,26]. Therefore, the 12-month interval used in this retrospective study was considered acceptable for evaluating agreement between imaging modalities.

The validity study aimed to evaluate the agreement and diagnostic performance of DXA-derived Cobb angle measurements relative to the radiographic reference standard. All radiographs were obtained in the standing posteroanterior (PA) position as part of routine clinical practice. The radiographic examinations included lumbar spine, thoracolumbar spine, or whole-spine radiographs, depending on clinical indication. Cobb angle measurements were performed using Synapse PACS version 5 by the principal investigator, who was trained in scoliosis assessment. DXA and radiographic measurements were performed at separate time points without reference to the corresponding measurements from the other imaging modality. Radiographic Cobb angle measurements were performed according to standard scoliosis assessment criteria and were not restricted to the vertebral levels visualized on the corresponding DXA images. Diagnostic performance of DXA-derived Cobb angle was assessed by determining sensitivity, specificity, positive predictive value, negative predictive value, likelihood ratios, and overall accuracy using radiographic findings as the reference standard.

### 2.4. Assessment of BMD

Bone mineral density was evaluated at the lumbar spine (L1 to L4), total hip, and femoral neck. The bone density values were used to calculate the T-score, using mean and standard deviation values for non-Hispanic white women aged 20–29 years from the NHANES (2005–2008). The T-scores at the lumbar spine, total hip, femoral neck, and the lowest T-score were then used to classify participants into three groups:

Normal: T-score ≥ −1.0;
Low bone mass (osteopenia): T-score between −2.5 and −1.0;
Osteoporosis: T-score ≤ −2.5.

### 2.5. Statistical Data Analysis

Statistical analysis was performed using STATA version 17 and Jamovi version 2.6.44. Baseline demographic characteristics were summarized using descriptive statistics, with continuous variables presented as mean ± standard deviation (SD) and categorical variables as counts and percentages. Differences between groups were assessed using the independent *t*-test for continuous variables and the Pearson’s chi-square test for categorical variables, with a *p*-value < 0.05 considered statistically significant. Available-case analysis (pairwise deletion) was used for variables with missing data. One participant had missing femoral neck and total hip T-score data and was excluded only from analyses involving those variables and the derived lowest T-score.

Univariate logistic regression analysis was performed to explore factors associated with lumbar scoliosis. Age, body mass index (BMI), and lumbar spine bone mineral density (BMD), selected based on clinical relevance, were included in the multivariable logistic regression model to assess independent associations. For the validity analysis, Cobb angle measurements obtained from lumbar DXA images were compared with those from spine radiographs, which served as the reference standard. Agreement between the two methods was evaluated using the intraclass correlation coefficient (ICC) and Bland–Altman analysis, with limits of agreement also assessed.

The diagnostic performance of the DXA-derived Cobb angle was evaluated by calculating sensitivity, specificity, positive predictive value, negative predictive value, likelihood ratios, and overall accuracy. In addition, receiver-operating characteristic (ROC) curve analysis was performed to assess the discriminative ability of DXA measurements, with the area under the curve (AUC) reported. The optimal cutoff value was determined using the Youden index, and the corresponding sensitivity and specificity were reported. Proportional bias was assessed by linear regression of the difference between DXA and radiographic Cobb angle measurements against the mean of the two measurements.

## 3. Results

### 3.1. Characteristics of the Study Population

A total of 267 eligible participants were identified. After exclusion of six participants with lumbar spinal instrumentation, 261 participants were included in the final analysis (Figure 1). The prevalence of DXA-detected lumbar scoliosis was 14.9% (39/261; 95% CI, 10.8–19.9%). Among the overall cohort, the mean age was 74.0 years (SD 8.8), the BMI was 24.1 kg/m^2^ (SD 3.9), and the mean Cobb angle was 5.3 degrees (SD 4.9), as summarized in Table 1.

Comparative analysis of demographic characteristics revealed that the scoliosis group was significantly older than the non-scoliosis group (*p* < 0.001). Conversely, no statistically significant differences were observed between the groups regarding BMI, BMD, or T-score classifications.

### 3.2. Comparison of Participants with and Without Radiographic Validation

Of the 261 participants, 67 had available radiographs and were included in the diagnostic validity analysis, whereas 194 participants did not undergo radiographic validation. Participants with radiographs were older and had lower femoral neck and total hip BMD values. They also had higher DXA Cobb angles and a greater prevalence of DXA-detected scoliosis than those without radiographs (28.4% vs. 10.3%, *p* < 0.001), suggesting that the validation subgroup may represent a clinically selected population. (Table 2). Therefore, the diagnostic performance results may not be fully generalizable to the overall DXA referral population.

### 3.3. Prevalence and Characteristics of DXA-Detected Lumbar Scoliosis

DXA-detected lumbar scoliosis was identified in 14.9% of participants (N = 39) based on lumbar DXA scans. Among participants with lumbar scoliosis, the mean Cobb angle was 14.4 ± 5.9 degrees. The DXA-derived characteristics of these scoliosis cases, including Cobb angle severity categories, curve apex level, curve direction, and vertebral rotation graded according to the Nash and Moe classification, are summarized in Table 3.

### 3.4. Logistic Regression Analysis

In the univariate analysis of risk factors associated with lumbar scoliosis, age was identified as the only significant factor. Increasing age was significantly associated with lumbar scoliosis (OR 1.09 per year, *p* < 0.001). BMI and BMD at the lumbar spine, femoral neck, and hip were not significantly associated with lumbar scoliosis, as shown in Table 4. When these factors were considered together in a multivariable model, age remained significantly associated with lumbar scoliosis (adjusted OR = 1.09, 95% CI 1.05–1.13, *p* < 0.001), whereas BMI and lumbar spine BMD were not significantly associated after adjustment (Table 5).

### 3.5. Reliability of Cobb Angle Measurements

Excellent intraobserver and interobserver reliability were observed for Cobb angle measurements on lumbar DXA images. The intraobserver intraclass correlation coefficient (ICC) was 0.97 (95% CI: 0.93–0.98), while the interobserver ICC was 0.96 (95% CI: 0.93–0.98). In addition, radiographic Cobb angle measurements demonstrated excellent interobserver reliability with an ICC(2,1) of 0.97 (95% CI: 0.94–0.99).

### 3.6. Validity Study of Scoliosis Assessment

**Figure 3:**
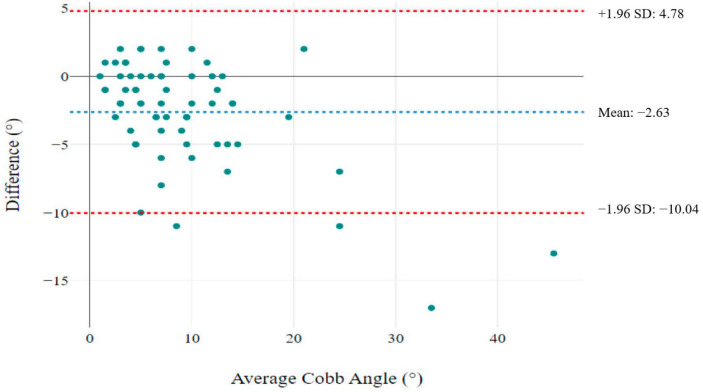
Bland–Altman plot shows the differences in Cobb angles obtained from assessments between lumbar DXA images and spine radiographs.

Among the 67 participants included in the diagnostic validity analysis, lumbar DXA and radiographic Cobb angle measurements demonstrated strong agreement, with an ICC of 0.91 (95% CI 0.73–0.96). The Bland–Altman plot indicated a mean difference of −2.63 degrees (95% CI [−3.55, −1.70]) between Cobb angles from lumbar DXA images and spine radiographs. Out of 67 participants, four (5.97%) were outside the limits of agreement, as shown in Figure 3.

The evaluation of Cobb angles from lumbar DXA images compared with spine radiographs showed a sensitivity of 62.1% (95% CI: 42.3–79.3%) and specificity of 97.4% (95% CI: 86.2–99.9%), with an overall accuracy of 82.1% (95% CI: 70.8–90.4%), as shown in Table 6. The cross-tabulation of DXA-derived scoliosis classification and radiographic findings is presented in Table 6. DXA correctly identified 18 of 29 radiographic scoliosis cases, while 11 cases were not identified by DXA. Only one participant without radiographic scoliosis was incorrectly classified as positive. All 11 false-negative cases had radiographic Cobb angles between 10° and 14°, indicating that missed cases were limited to mild curves near the diagnostic threshold. Among these 11 false-negative cases, 5 (45.5%) had radiographic curves extending above T12, beyond the superior field of view of lumbar DXA imaging. This finding suggests that differences in anatomical coverage between DXA and radiographs may have contributed to discordant classifications in some participants. Diagnostic performance metrics derived from this classification are summarized in Table 7.

Proportional bias analysis demonstrated a significant negative association between measurement difference and mean Cobb angle (slope = −0.29, *p* < 0.001), indicating that the degree of underestimation by DXA increased with greater curve magnitude. This finding suggests that agreement between DXA and radiographic measurements was not uniform across the full range of Cobb angles.

### 3.7. Receiver-Operating Characteristic (ROC) Analysis

**Figure 4:**
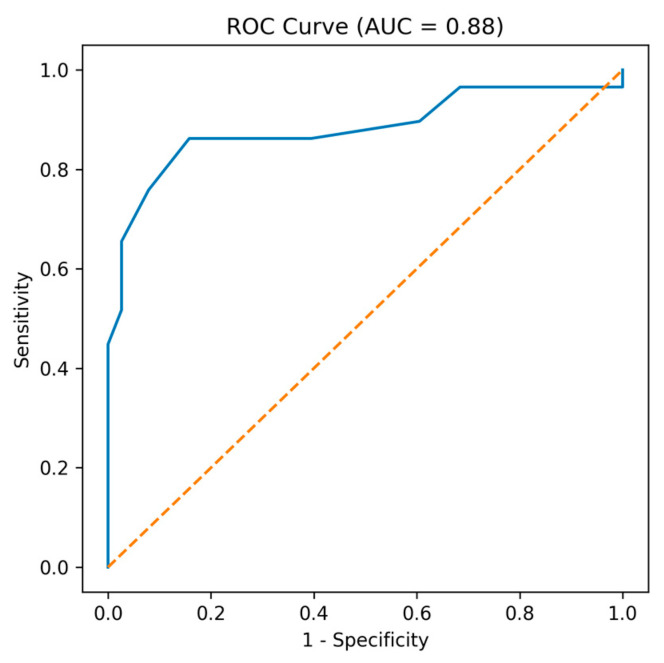
Receiver-operating characteristic (ROC) curve of DXA-derived Cobb angle for detecting lumbar scoliosis using radiographic findings as the reference standard. The area under the curve (AUC) was 0.88.

ROC analysis showed that DXA-derived Cobb angle demonstrated good discriminative ability for detecting scoliosis, with an AUC of 0.88 (95% CI: 0.79–0.98). An exploratory cutoff of 6.5 degrees yielded the highest Youden index, corresponding to a sensitivity of 86.2% (95% CI: 68.3–96.1%) and specificity of 84.2% (95% CI: 68.8–94.0%). Compared with the conventional 10-degree threshold, the 6.5-degree cutoff increased sensitivity while reducing specificity. The ROC curve is shown in Figure 4.

## 4. Discussion

This study aimed to investigate the prevalence of DXA-detected lumbar scoliosis and associated risk factors among postmenopausal women over 50 years of age who underwent DXA. The findings revealed a DXA-detected lumbar scoliosis prevalence of 14.9% (95% CI, 10.8–19.9%). Previous studies have reported substantial variation in scoliosis prevalence across different populations and study methodologies. Our findings are consistent with those reported by Urrutia et al. [9], who reported a prevalence of 12.9% in a similar population. Rubin et al. [7] reported a higher prevalence of 25.2% in postmenopausal women and demonstrated increasing prevalence with advancing age, which is consistent with the significant association between age and lumbar scoliosis observed in the present study.

Increasing age was the only variable significantly associated with lumbar scoliosis (OR 1.09 per year, 95% CI 1.04–1.13, *p* < 0.001). After adjustment, age remained the only independent factor associated with lumbar scoliosis in this cohort, supporting the role of degenerative changes in its development. This aligns with findings from previous studies by Hong, Rubin, Urrutia and Xu [4,7,9,10], which reported a positive association between increasing age and the prevalence of lumbar scoliosis. Interestingly, the study found no significant correlation between body mass index (BMI) and lumbar scoliosis, which differs from the findings of Urrutia et al., who found that lower BMI was associated with lumbar scoliosis [9]. This difference could be attributed to the smaller sample size in this study compared to Urrutia’s.

Additionally, the relationship between BMD measurements at various sites, L1–L4, total hip, and femoral neck, and lumbar scoliosis was also insignificant. This finding corroborates the conclusions of Rubin and Urrutia [7,9], reinforcing the notion that low BMD does not necessarily predispose individuals to lumbar scoliosis. Interpretation of lumbar spine BMD in individuals with scoliosis should nevertheless be approached with caution, as degenerative changes, vertebral rotation, osteophytes, and endplate sclerosis may influence DXA measurements. Hip and femoral neck BMD may therefore provide more robust estimates of skeletal status in the presence of spinal deformity.

The analysis of Cobb angles from DXA imaging revealed a mean difference of −2.63 degrees when compared to standard radiographs, highlighting a potential underestimation of the curvature in DXA images. This could be due to differences in positioning, as DXA scans are conducted in a supine position without weight bearing, potentially resulting in reduced curvature compared to standing radiographic views. Furthermore, the visibility limitations of the DXA scans, which are confined to the T12-L5 vertebrae, compared to radiographs that can assess the entire spinal column, may also contribute to these differences.

Although the ICC indicated strong agreement, clinically meaningful disagreement may still occur near the diagnostic threshold. The mean difference of −2.63° and the observed false-negative cases highlight that agreement statistics should be interpreted alongside Bland–Altman findings when evaluating diagnostic classification. A proportional bias analysis demonstrated that the degree of underestimation increased with greater curve magnitude. This finding is consistent with the observed negative mean difference and may partly explain the larger discrepancies observed in participants with higher Cobb angles.

Nevertheless, Cobb angle measurements obtained from lumbar spine DXA scans showed strong agreement with lumbar spine radiographs, the reference standard for scoliosis assessment (ICC = 0.91, 95% CI 0.73–0.96). This is in line with prior studies by Pappou, Schell, and colleagues [15,16], which have reported that DXA-derived measurements are comparable to radiographs. Taken together, these findings support the reliability and overall validity of DXA-derived Cobb angle measurements for opportunistic assessment of lumbar scoliosis, while acknowledging some degree of underestimation relative to standing radiographs.

The findings of the present study are consistent with those of Jamaludin et al. [27], who demonstrated good diagnostic performance of lumbar spine DXA for detecting lumbar scoliosis compared with radiographs. Similarly, our results showed high specificity and positive predictive value with a low false-positive rate, supporting the use of lumbar spine BMD scan images for opportunistic case detection. While false-positive findings in both studies were mainly attributed to patient positioning and measurement-related errors leading to overestimation of Cobb angles on DXA images, the present study demonstrated a small negative mean difference, suggesting a tendency toward underestimation on average.

The ROC findings provide additional context for interpreting the diagnostic performance of DXA. While the traditional 10-degree cutoff offers excellent specificity, ROC analysis suggested that a lower DXA Cobb angle threshold may improve sensitivity for opportunistic scoliosis detection. An exploratory cutoff of 6.5 degrees yielded the highest Youden index, which increased sensitivity to 86.2% at the expense of reduced specificity (84.2%) compared with the conventional 10-degree threshold. This lower threshold may partially reflect the tendency of DXA to underestimate Cobb angle measurements compared with standing radiographs. However, this finding was derived from a relatively small validation cohort and should be considered exploratory until externally validated in independent populations.

Although sensitivity was modest, DXA demonstrated very high specificity and positive predictive value. Among the 29 participants with radiographic scoliosis, 11 had DXA Cobb angles below 10 degrees and were classified as false negatives. The negative mean difference observed in the Bland–Altman analysis suggests that DXA tends to underestimate Cobb angle measurements compared with standing radiographs. This underestimation may have contributed to false-negative classification, particularly in borderline cases near the 10-degree diagnostic threshold. Notably, all false-negative cases had mild radiographic scoliosis, with Cobb angles ranging from 10° to 14°. In addition, several false-negative cases involved curves extending above T12, suggesting that the limited field of view of lumbar DXA may have contributed to the underestimation of curve severity in some participants. Accordingly, some false-negative classifications may reflect anatomical coverage limitations of DXA rather than solely measurement inaccuracy, and a negative DXA result cannot reliably exclude scoliosis. These findings suggest that DXA may be more suitable as an opportunistic case detection or rule-in tool rather than a standalone rule-out screening test. As DXA is already routinely performed for osteoporosis assessment in postmenopausal women, its value lies in opportunistic case detection without additional radiation exposure, cost, or separate imaging appointments.

Furthermore, although the interval between DXA and radiographs reached up to 12 months, this duration is generally acceptable because degenerative lumbar scoliosis progresses slowly and measurable progression often requires several years of follow-up [3,14,25,26]. Given the generally slow progression of degenerative lumbar scoliosis, the impact of this interval on diagnostic classification was likely limited, although it cannot be completely excluded.

This study faced several limitations. First, the maximum 12-month interval between imaging modalities may introduce variability due to potential spinal changes over time. However, degenerative lumbar scoliosis generally progresses slowly, and the excellent ICC and high specificity observed in this study suggest that Cobb angle measurements remained robust for opportunistic case detection despite this timeframe. Second, the DXA scans were performed in a supine position, which does not replicate weight-bearing conditions and may contribute to underestimation of Cobb angle measurements and false-negative classification due to reduced curve magnitude. Third, the limited field of view of lumbar DXA images may result in incomplete visualization of spinal curvature, particularly when scoliosis extends beyond the vertebral levels captured on DXA [16]. Because radiographic measurements were based on the full visible curve rather than the DXA field of view, some false-negative cases may have been influenced by the limited anatomical coverage of lumbar DXA images. Fourth, the relatively small sample size may have limited the ability to detect statistically significant associations between lumbar scoliosis and factors such as BMI and BMD. In addition, the subgroup of participants who underwent spine radiographs may represent a clinically selected population rather than a true screening population, which could introduce selection and verification bias in the diagnostic validity analysis. Clinical indications for radiographic examinations were not systematically available in this retrospective study, limiting further evaluation of factors associated with inclusion in the validation subgroup. Therefore, the diagnostic performance estimates should be interpreted with caution and may not be fully generalizable to the overall DXA referral population. Furthermore, this was a single-center study conducted in a Thai population, which may limit the generalizability of the findings to other ethnic, racial, or geographic populations. Future studies with larger sample sizes may benefit from age-stratified analyses to allow more direct comparison with previously published prevalence studies. Despite these limitations, the high specificity and diagnostic accuracy observed support the clinical utility of DXA as a viable tool for opportunistic case detection of lumbar scoliosis.

## 5. Conclusions

In conclusion, this study demonstrates a 14.9% prevalence of DXA-detected lumbar scoliosis among postmenopausal women undergoing DXA assessment, with increasing age identified as the only significant factor associated with scoliosis. Despite the potential for mild underestimation related to supine positioning and limited anatomical coverage, DXA-derived Cobb angle measurements showed excellent reliability and strong agreement with standard radiographs. These findings support the potential role of DXA as a tool for opportunistic case detection of lumbar scoliosis during routine BMD assessment. Given its high specificity but modest sensitivity, DXA appears more useful for identifying likely scoliosis than for excluding disease. Future studies with larger and more diverse populations are warranted to further validate these findings and clarify the clinical implications of opportunistic scoliosis detection on DXA images.

## Figures and Tables

**Table 1:** Demographic Characteristics and Comparisons Between Scoliosis and Non-Scoliosis Groups in Postmenopausal Women (N = 261).

Characteristics	Overall (N = 261)	Scoliosis N = 39 (14.9%)	Non-Scoliosis N = 222 (85.1%)	*p*-Value
Age (years)	74.0 ± 8.8	79.3 ± 8.7	73.1 ± 8.4	<0.001
BMI (kg/m^2^)	24.1 ± 3.9	24.1 ± 4.2	24.1 ± 3.9	0.977
Mean Cobb angle (°)	5.3 ± 4.9	14.4 ± 5.9	3.7 ± 2.2	<0.001
Lumbar spine BMD (g/cm^2^)	0.826 ± 0.152	0.826 ± 0.185	0.826 ± 0.146	1.000
Femoral neck BMD (g/cm^2^)	0.607 ± 0.121	0.593 ± 0.121	0.610 ± 0.121	0.410
Total hip BMD (g/cm^2^)	0.747 ± 0.132	0.723 ± 0.141	0.752 ± 0.130	0.209
Lumbar spine T-score				0.270
Normal	56 (21.5%)	11 (28.2%)	45 (20.3%)	
Low bone mass	80 (30.7%)	8 (20.5%)	72 (32.4%)	
Osteoporosis	125 (47.9%)	20 (51.3%)	105 (47.3%)	
Femoral neck T-score *				0.860
Normal	32 (12.3%)	4 (10.3%)	28 (12.6%)	
Low bone mass	89 (34.1%)	12 (30.8%)	77 (34.7%)	
Osteoporosis	139 (53.3%)	23 (58.9%)	116 (52.3%)	
Total hip T-score *				0.542
Normal	55 (21.1%)	8 (20.5%)	47 (21.2%)	
Low bone mass	116 (44.4%)	14 (35.9%)	102 (45.9%)	
Osteoporosis	89 (34.1%)	17 (43.6%)	72 (32.4%)	
Lowest T-score *				0.900
Normal	21 (8.0%)	3 (7.7%)	18 (8.1%)	
Low bone mass	74 (28.4%)	10 (25.6%)	64 (28.8%)	
Osteoporosis	165 (63.2%)	26 (66.7%)	139 (63.1%)	

* Missing data (N = 1).

**Table 2:** Comparison of participants with and without radiographs (n = 261).

Characteristics	With Radiographs (n = 67)	Without Radiographs (n = 194)	*p*-Value
Age (years)	76.0 ± 8.7	73.5 ± 8.7	0.045
BMI (kg/m^2^)	24.3 ± 4.3	24.0 ± 3.8	0.578
Lumbar spine BMD (g/cm^2^)	0.810 ± 0.167	0.831 ± 0.146	0.317
Femoral neck BMD (g/cm^2^) *	0.572 ± 0.109	0.620 ± 0.123	0.005
Total hip BMD (g/cm^2^) *	0.716 ± 0.149	0.758 ± 0.125	0.025
DXA Cobb angle (°)	7.55 ± 6.61	4.58 ± 3.86	<0.001
DXA-detected scoliosis, n (%)	19 (28.4%)	20 (10.3%)	<0.001

Data are presented as mean ± SD unless otherwise indicated. DXA-detected scoliosis was defined as a Cobb angle ≥10° on lumbar DXA images. * Missing data (N = 1).

**Table 3:** DXA-derived characteristics of participants with lumbar scoliosis (N = 39).

	Scoliosis Patients (%)
Cobb angle	
10–19°	33 (84.6)
20–29°	5 (12.8)
30–39°	1 (2.6)
Curve apex	
L1	3 (7.7)
L2	12 (30.8)
L3	22 (56.4)
L4	2 (5.1)
Curve direction	
Left	21 (53.8)
Right	18 (46.2)
Nash–Moe grade *	
1	27 (69.2)
2	11 (28.2)
3	1 (2.6)

* No participants demonstrated Nash–Moe grade 0 or grade 4 rotation.

**Table 4:** Results of univariate analysis of lumbar scoliosis in the study population.

	Odds Ratio (95% CI)	*p*-Value
Age (per year)	1.09 (1.04–1.13)	<0.001
BMI (kg/m^2^)	1.00 (0.92–1.09)	0.977
Lumbar spine BMD (per 0.1 g/cm^2^ increase)	1.00 (0.80–1.25)	1.000
Femoral neck BMD (per 0.1 g/cm^2^ increase)	0.89 (0.66–1.18)	0.408
Total hip BMD (per 0.1 g/cm^2^ increase)	0.84 (0.65–1.10)	0.209

**Table 5:** Multivariable logistic regression analysis for factors associated with lumbar scoliosis.

	Adjusted OR (95% CI)	*p*-Value
Age (per year)	1.09 (1.05–1.13)	<0.001
BMI (kg/m^2^)	0.98 (0.89–1.08)	0.706
Lumbar spine BMD (per 0.1 g/cm^2^ increase)	1.07 (0.85–1.35)	0.568

**Table 6:** Cross-tabulation of DXA-derived scoliosis classification and radiographic reference standard (n = 67).

	Radiographic Scoliosis (+)	Radiographic Scoliosis (−)	Total
DXA scoliosis (+)	18	1	19
DXA scoliosis (−)	11	37	48
Total	29	38	67

**Table 7:** Diagnostic performance of lumbar DXA images for detecting scoliosis using radiographs as the reference standard.

	Value (95% CI)
Sensitivity (%)	62.1 (42.3–79.3)
Specificity (%)	97.4 (86.2–99.9)
PPV (%)	94.7 (71.8–99.2)
NPV (%)	77.0 (67.8–84.3)
LR+	23.9 (3.34–166.6)
LR−	0.4 (0.24–0.62)
Accuracy (%)	82.1 (70.8–90.4)

PPV = Positive Predictive Value, NPV = Negative Predictive Value, LR+ = Positive Likelihood Ratio, LR− = Negative Likelihood Ratio.

## Data Availability

The original contributions presented in this study are included in the article. Further inquiries can be directed to the corresponding author.
